# Finding function: evaluation methods for functional genomic data

**DOI:** 10.1186/1471-2164-7-187

**Published:** 2006-07-25

**Authors:** Chad L Myers, Daniel R Barrett, Matthew A Hibbs, Curtis Huttenhower, Olga G Troyanskaya

**Affiliations:** 1 Department of Computer Science, Princeton University, Princeton, NJ 08544, USA; 2 Lewis-Sigler Institute for Integrative Genomics, Princeton University, Princeton NJ, 08544, USA

## Abstract

**Background:**

Accurate evaluation of the quality of genomic or proteomic data and computational methods is vital to our ability to use them for formulating novel biological hypotheses and directing further experiments. There is currently no standard approach to evaluation in functional genomics. Our analysis of existing approaches shows that they are inconsistent and contain substantial functional biases that render the resulting evaluations misleading both quantitatively and qualitatively. These problems make it essentially impossible to compare computational methods or large-scale experimental datasets and also result in conclusions that generalize poorly in most biological applications.

**Results:**

We reveal issues with current evaluation methods here and suggest new approaches to evaluation that facilitate accurate and representative characterization of genomic methods and data. Specifically, we describe a functional genomics gold standard based on curation by expert biologists and demonstrate its use as an effective means of evaluation of genomic approaches. Our evaluation framework and gold standard are freely available to the community through our website.

**Conclusion:**

Proper methods for evaluating genomic data and computational approaches will determine how much we, as a community, are able to learn from the wealth of available data. We propose one possible solution to this problem here but emphasize that this topic warrants broader community discussion.

## Background

Recent advances in experimental methods have enabled the development of functional genomics, a genome-wide approach to understanding the inner workings of a cell. While such large-scale approaches will undoubtedly be instrumental in extending our knowledge of molecular and cellular biology, they produce enormous amounts of heterogeneous data of varying relevance and reliability. A key challenge in interpreting these data is separating accurate, functionally relevant information from noise.

Here we focus on using noisy genomic datasets to associate uncharacterized genes or proteins with biological processes. Recent literature on protein function prediction focuses on integrating multiple sources of evidence (e.g. physical interactions, genetic interaction, gene expression data) to assign proteins to processes [1-4] or to predict functional associations or interactions between related proteins [5-10]. Individual high-throughput datasets are typically noisy, but effective integration can yield precise predictions without sacrificing valuable information in the data. All of these methods require a gold standard, which is a trusted representation of the functional information one might hope to discover. Such a standard, coupled with an effective means of evaluation, can be used to assess the performance of a method and serves as a basis for comparison with existing approaches. Beyond methods for predicting protein function or interactions, evaluation against gold standards can be used to directly measure the quality of a single genomic dataset, a necessary step in developing and validating new experimental technology.

**Figure 1:**
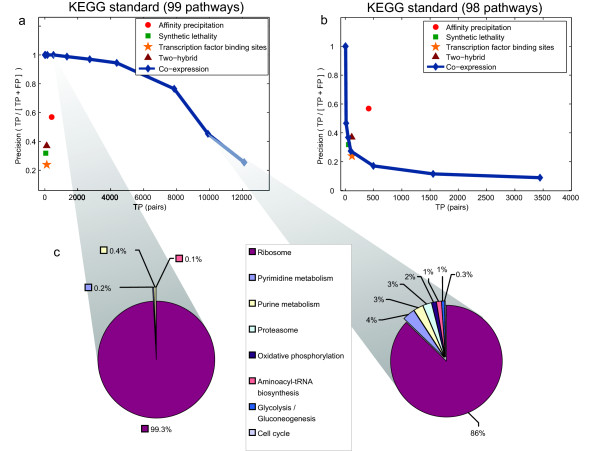
**Inconsistencies in evaluation due to process-specific variation in performance**. (**a **and **b**) Comparative functional evaluation of several high-throughput datasets based on a KEGG-derived gold standard. The evaluation pictured in (**b**) is identical to that in (**a**) except that one of ninety-nine KEGG pathways was excluded from the analysis ("Ribosome," sce03010). Gold standard positives were obtained by considering all protein pairs sharing a KEGG pathway annotation as functional pairs, while gold standard negatives were taken to be pairs of proteins occurring in at least one KEGG pathway but with no co-annotation. Performance is measured as the trade-off between precision (the proportion of true positives to total positive predictions) and true positive pairs. For the evaluation in (**b**), both precision and sensitivity drop dramatically for co-expression data. (**c**) Composition of correctly predicted positive protein-protein relationships at two different choices of precision-recall. Of the 0.1% most co-expressed pairs, 99.3% of the true positive pairs (842 of 848) are due to co-annotation to the ribosome pathway (left pie chart). This bias is less pronounced at lower precision but still present. Of the 1% most co-expressed pairs, 86% of the true positive pairs (8500 of 9900) are due to co-annotation to the ribosome pathway (right pie chart).

We have undertaken a study of proposed standards and approaches to evaluation of functional genomic data and highlight a number of important issues. We find that current approaches are inconsistent, making reported results incomparable, and often biased in such a way that the resulting evaluation cannot be trusted even in a qualitative sense. One specific problem we identify is substantial functional biases in typical gold standard datasets. We demonstrate this problem by evaluating several functional genomic datasets using the Kyoto Encyclopedia of Genes and Genomes (KEGG)[11] as a gold standard (Fig. 1), as is commonly employed in the literature (e.g. [7,12]). A naïve evaluation in this manner identifies co-expression data as by far the most sensitive and specific genome-scale functional genomic data type (Fig. 1a). However, this apparent superior performance is due to characteristics of a single pathway; when the ribosome (1 out of 99 total KEGG pathways) is removed from the gold standard, co-expression becomes one of the least informative datasets (Fig. 1b). In addition to such substantial functional biases, we find that commonly used gold standards are highly inconsistent even for comparative evaluations and that most current evaluation methodologies yield misleading estimates of accuracy.

In this paper, we describe these problems with current evaluation standards with the hope of instigating a community dialog on proper approaches to comparing genomic data and methods. As noted above, there are two typical approaches to using genomic data for analyzing protein function: methods that directly associate proteins with particular processes or functional classes, and methods that focus on predicting functional associations or interactions between pairs of proteins. We focus our attention toward standards for the latter, evaluating pairwise associations between genes produced by either experimental or computational techniques. Many of the problems we describe, however, apply to both approaches, and we suggest an alternative standard for evaluation that is appropriate in both settings. We provide both a trusted set of functional associations between proteins as well as a specific set of biological processes that maps proteins to well-defined functional classes. Both standards are based on curation by a panel of biological experts. Furthermore, we propose several guidelines for using these standards to perform accurate evaluation of methods and data. The resulting evaluation framework can be used to directly measure and compare the functionally relevant information present in raw high-throughput datasets as well as to evaluate or train computational genomics methods.

Our gold standard and evaluation methodology have been implemented in a web-based system [13] to facilitate community use for comparison among published datasets or methods. We demonstrate the use of our approach on genomic data from *Saccharomyces cerevisiae*. Accurate evaluation methods are particularly critical for this model organism, because yeast is widely used as a platform for the development of both high-throughput experimental techniques and computational methods. However, the weaknesses we identify in existing evaluation methodologies as well as the solution we propose are applicable to data from other model organisms and humans.

## Results and discussion

We first discuss commonly used gold standards and several fundamental issues with current approaches to evaluation of functional genomic data and methods. To address these problems, we propose a new gold standard based on expert curation and recommend appropriate uses of the standard that ensure accurate evaluation. Finally, we describe a web-based implementation of our evaluation framework, which is available for public use by computational and experimental biologists.

### Challenges to effective functional evaluation

#### Existing gold standards

A number of different gold standards for evaluating yeast functional genomic data or methods have been proposed in the literature. Each standard generally consists of sets of gene or protein pairs grouped as either "positive" or "negative" examples. This is due in large part to the fact that some high throughput data takes the form of associations between genes or gene products (e.g. physical or genetic interactions). Furthermore, a pairwise approach to analysis is a natural way to view biological systems, which are composed of networks, or groups of interactions between gene products. Although this is a commonly adopted approach, others have trained classifiers for specific functional classes where individual proteins or genes are directly associated with functional classes or processes [1,4]. While we focus on data and methods for pairwise associations between proteins here, many of the issues described are equally problematic for such non-pairwise approaches, and we propose an alternative gold standard appropriate for both settings (see details in "Defining a new gold standard" in Methods).

Most functional genomics evaluations derive gold standard positives from functional classification schemes that capture associations of genes or proteins with specific biological processes as reported in the literature [7,10,12,14-18]. Such classifications are available from multiple sources including the Gene Ontology (GO)[19] (and associated annotation repositories such as the *Saccharomyces *Genome Database)[20], KEGG [11], the Munich Information Center for Protein Sequences (MIPS) [21], and the Yeast Protein Database (YPD) [22]. A common source of gold standard negatives is cellular localization data [6,7,23,24]. Most of these methods utilize a localization study in which 75% of the yeast proteome was GFP-tagged and classified into 22 different cellular compartments [25] and they assume that two proteins localizing to distinct compartments do not interact. Random pairs of proteins sampled from the proteome provide another common gold-standard negative, relying on the assumption that the expected number of functionally related or interacting pairs is much less than the total number of possible pairwise protein-protein combinations [5,26,27].

#### Inconsistencies among and within different standards

**Figure 2:**
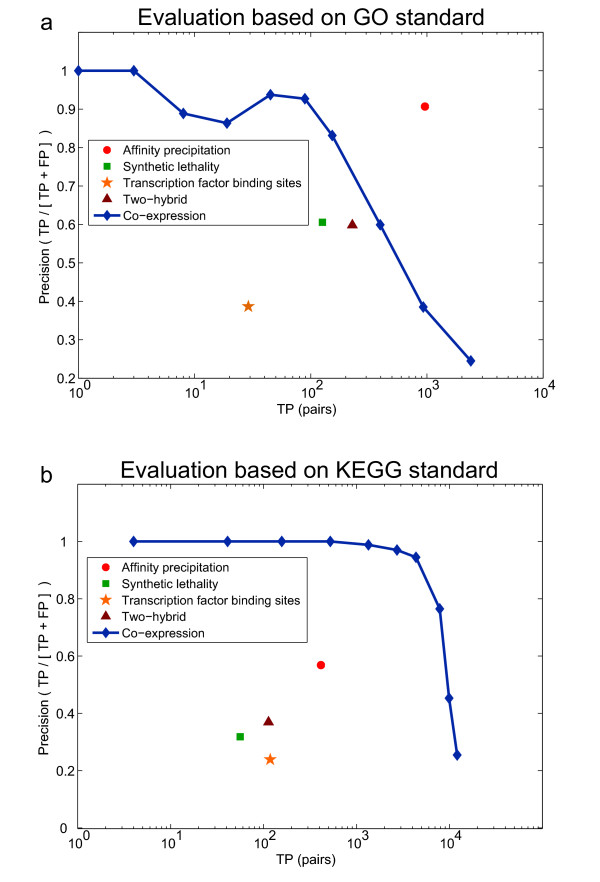
**Comparison of functional genomic data evaluation on GO and KEGG gold standards**. (**a**) Comparative functional evaluation of several high-throughput evidence types based on a typical Gene Ontology (GO) gold standard. Positive pairs were obtained by finding all protein pairs with co-annotations to terms at depth 8 or lower in the biological process ontology. Negative pairs were generated from protein pairs whose most specific co-annotation occurred in terms with more than 1000 total annotations. (**b**) Evaluation of the same data against a KEGG-based gold standard. Gold standard positives were obtained by considering all protein pairs sharing a KEGG pathway annotation as functional pairs, while gold standard negatives were taken to be pairs of proteins occurring in at least one KEGG pathway but with no co-annotation. There are several serious inconsistencies between the two evaluations. In addition to vastly different estimates of the reliability of co-expression data, other evidence types change relative positions. For instance, transcription factor binding site predictions appear competitive with both two-hybrid and synthetic lethality in the KEGG evaluation, but are substantially out-performed in the GO evaluation. These inconsistencies between the two gold standards demonstrate the need for a common, representative evaluation framework.

Perhaps the most apparent issue with functional genomic evaluation arises from the diversity of possible standards and lack of agreement among them. It has been noted that gold standard positive pairs derived from KEGG, MIPS, and GO biological process ontology show little overlap [28]. We find even less agreement among gold standards for physical interactions predictions, which are usually based on small interaction datasets obtained from protein-protein interaction databases such as the Database of Interacting Proteins (DIP)[29], the General Repository for Interaction Datasets (GRID)[30], or the Biomolecular Interaction Network Database (BIND) [31]. However, the more alarming problem is that even the *relative *performance of methods or datasets evaluated against these standards is not consistent. For example, using both the biological process GO and the KEGG pathways gold standard to evaluate the relative performance of commonly used data sets produces strikingly different results (Fig. 2). This difference is likely due to the nature of the biological relationships each standard is trying to capture or simply variation in which specific proteins are present in the classification scheme or interaction dataset. Although each standard is correctly evaluating some aspect of the data, without a common, representative evaluation framework, the community cannot assess the relative performance of novel methods or high-throughput techniques.

**Figure 3:**
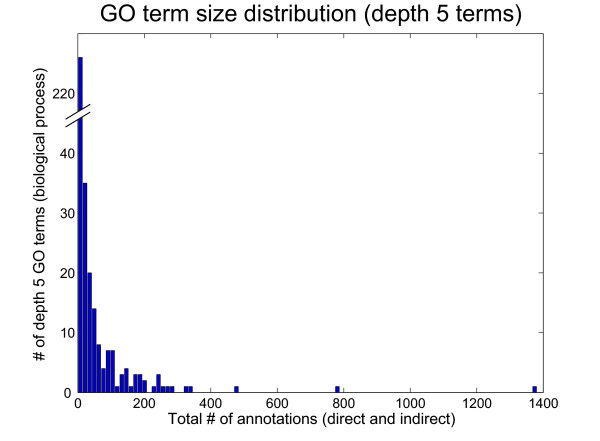
**Size distribution of depth 5 biological process GO terms (*S. cerevisiae*)**. Depth and size are commonly used metrics for assessing the biological specificity of GO terms, a necessary step in creating a functional gold standard from the ontology. Here, the number of direct and indirect annotations was counted for each depth 5 GO term and counts were binned to obtain a histogram of sizes for depth 5 GO terms. This reveals a wide range of sizes for terms at the same depth (from 0 annotations to 1381 annotations), suggesting size and depth are not capturing the same notion of specificity, and that likely neither is an appropriate measure for true biological specificity. A sampling of the largest and smallest depth 5 GO terms is shown in Table 1.

In addition to substantial inconsistencies among existing gold standards, variation in biological specificity within each standard has also impaired previous evaluation methods. Standards based on biological ontologies (e.g. GO or the MIPS Functional Catalogue) classify proteins at a broad range of resolutions (e.g. metabolism vs. carbohydrate metabolism). Although these ontologies can provide a powerful framework for defining a gold standard, there are a few caveats. A typical approach for using GO has been to pick a particular depth in the hierarchy below which term co-annotations imply gold standard positives. However, terms at the same level can vary dramatically in biological specificity [32] (Fig. 3 and Table 1). For example, at a depth of 5 in the biological process GO, the term "regulation of sister chromatid cohesion" (GO:0007063) with a single indirect gene product annotation appears alongside a much more general term "cellular protein metabolism" (GO:0044267), which has 1381 annotations. Widely varying degrees of specificity in a gold standard not only complicate evaluation methods but can also appear as inconsistencies in the data when training machine learning algorithms, which can result in poor performance.

#### Functional biases in prediction performance

The majority of current evaluation approaches are performed without regard to which biological processes are represented in the set of true positives (correctly predicted examples), and thus they are often unknowingly skewed toward particular processes. We illustrate this bias with an example using the KEGG pathways gold standard to evaluate genomic data (Fig. 1). In this evaluation, the estimated reliability of microarray co-expression drops dramatically when a single pathway ("Ribosome" or sce3010) is excluded from the analysis. The substantial drop in precision suggests that a large fraction of the true positives predicted by co-expression are exclusively ribosome relationships. In fact, of the positive examples in the 1% most co-expressed pairs, 86% (~8500 of 9900) are due to co-annotation to the ribosome pathway. This bias becomes even more pronounced at higher co-expression level cutoffs: of the 0.1% most co-expressed positive pairs, 99% (842 of 848) are from the ribosome pathway. We find a similar bias in evaluations using the GO and MIPS gold standards.

Thus, the traditional approach of using a general ROC curve (or related measure) without regard to which processes are represented can be misleading (see Methods for a discussion of ROC curves). This is particularly true when the data or computational predictions have process-dependent reliability as is often the case with genomic or proteomic data. The problem is magnified when the gold standard examples themselves are heavily skewed towards specific functional categories. While the general precision-recall characteristics such as those portrayed in Figure 1 are technically correct, they generalize poorly to non-ribosomal protein relationships. Thus, such an evaluation would be misleading for a scientist hoping to use these data to generate new hypotheses about proteins unrelated to the ribosome. We address this problem in our process-specific evaluation framework.

#### Gold standard negatives

Another shortcoming of current standards for gene/protein function prediction is the nature of the gold standard negative examples. In yeast, one proposed source of gold standard negatives is based on protein localization data [23,25] because pairs of proteins localizing to different cellular compartments are highly enriched for non-interacting proteins. However, localization data is likely not *representative *of "typical" unrelated protein pairs. For instance, Ben-Hur and Noble found the performance of SVM classifiers trained with localization negatives artificially inflated because this negative set is composed entirely of high-confidence pairs [5,33]. Using such a non-representative "easy" set of negatives will overestimate prediction accuracy, and the resulting classifier will generalize poorly to real biological problems.

Thus, although protein localization data is a strong negative indicator of functional relationships or interactions, we caution against its use as a general negative gold standard. This is particularly problematic for higher-level questions such as function prediction, because proteins co-involved in some biological processes span cellular compartments. Perhaps a safer role for localization data is as the input to computational approaches. We suggest an alternative negative standard based on the biological process Gene Ontology that can provide representative negative examples (see "Suggestions for representative functional evaluation of data and methods").

#### Relative size of gold standard positive/negative sets

A final issue common among many evaluation standards in the literature is the relative size of the positive and negative example sets. The expected number of proteins involved in any particular biological process is a small percentage of the proteome, which should be reflected in evaluation standards. This imbalance is particularly problematic in methods based on pairwise associations between proteins, where the expected number of protein pairs sharing functional relationships is an even smaller fraction of all possible protein combinations. For instance, of the 18 million possible protein pairs in yeast, it is expected that less than 1 million are functionally related. This large difference makes the typical reporting of sensitivity and specificity misleading. For instance, a recently published method for predicting protein-protein interactions from several genomic features showed seemingly impressive 90% sensitivity and 63% specificity in evaluations [24], but would make correct predictions only 1 out of every 9 times when applied on a whole-genome scale, rendering the method impractical in many experimental contexts (details in additional file 3: Supplementary discussion).

Given this imbalance, an appropriate measure of functional relevance of genomic data or predictions is the precision or positive predictive value (PPV)(TPTP+FP)
 MathType@MTEF@5@5@+=feaafiart1ev1aaatCvAUfKttLearuWrP9MDH5MBPbIqV92AaeXatLxBI9gBaebbnrfifHhDYfgasaacH8akY=wiFfYdH8Gipec8Eeeu0xXdbba9frFj0=OqFfea0dXdd9vqai=hGuQ8kuc9pgc9s8qqaq=dirpe0xb9q8qiLsFr0=vr0=vr0dc8meaabaqaciaacaGaaeqabaqabeGadaaakeaadaqadiqaamaalaaabaGaemivaqLaemiuaafabaGaemivaqLaemiuaaLaey4kaSIaemOrayKaemiuaafaaaGaayjkaiaawMcaaaaa@361B@[23]. This measure rewards methods that generate firm positive predictions, without regard to the accuracy of negative predictions, which are less helpful in guiding laboratory experiments. Direct application of precision may be misleading, though, because this measure is only correct under the assumption that the ratio of positive to negative examples in the gold standard matches that in the application domain. If the ratio of positive to negatives in the gold standard is much larger than in whole-genome data, as is often the case in published evaluations, then the number of false positive predictions will be small and will artificially inflate the precision statistic. For instance, the 90%-63% sensitivity-specificity example above used an approximately equal number of positive and negative examples (1500 and 2000 respectively), leading to 65% precision. However, application of this method on a whole-genome scale, where the ratio of positive to negative examples is roughly 20 times smaller, would lead to an expected precision of just 11% (details in additional file 3: Supplementary discussion).

To avoid such misleading evaluations, the balance of positives and negatives in the gold standard should match that of the application domain as closely as possible. Precision, or PPV, then becomes a direct, representative measure of how well one could expect a dataset or method to perform on whole-genome tasks. Of course, precision alone does not convey all of the important information, only the *quality *of the predictions made by a dataset or method. It must be reported in tandem with some measure of the *quantity *of true predictions made. A standard measure for this is the recall, or sensitivity (TPTP+FN)
 MathType@MTEF@5@5@+=feaafiart1ev1aaatCvAUfKttLearuWrP9MDH5MBPbIqV92AaeXatLxBI9gBaebbnrfifHhDYfgasaacH8akY=wiFfYdH8Gipec8Eeeu0xXdbba9frFj0=OqFfea0dXdd9vqai=hGuQ8kuc9pgc9s8qqaq=dirpe0xb9q8qiLsFr0=vr0=vr0dc8meaabaqaciaacaGaaeqabaqabeGadaaakeaadaqadiqaamaalaaabaGaemivaqLaemiuaafabaGaemivaqLaemiuaaLaey4kaSIaemOrayKaemOta4eaaaGaayjkaiaawMcaaaaa@3617@, which is what is used in our evaluation framework (for more details, see Methods).

### Suggestions for representative functional evaluation of data and methods

In light of these problems with current gold standards and approaches to evaluation, we have compiled a new functional genomics gold standard and suggest several strategies for accurate comparative evaluation of genomic datasets and methods.

#### Defining a new gold standard

As discussed previously, a major issue with the current state of the community is inconsistency among the variety of standards used. Evaluations based on different standards (e.g. derived from KEGG versus GO) are often not comparable, even in a qualitative sense. Deriving a standard from these hierarchies is further complicated due to varying levels of biological specificity of curated biological knowledge. Furthermore, each of the sources of curated information has inherent functional biases that can lead to incorrect estimates of accuracy.

To develop a unified standard for general application in functional genomics, several key criteria must be met. The standard must be cross-organismal to ensure relevance to a broad audience. Secondly, the standard should cover a wide variety of biological functions or processes to facilitate comprehensive evaluations. Finally, the standard should adapt quickly as biological knowledge expands. Although there are several sources of annotation that satisfy these criteria to varying extents (eg. KEGG, MIPS, and GO), GO is arguably the best option to serve as a foundation for the standard, as it is well-curated and was designed for complete coverage.

**Figure 4:**
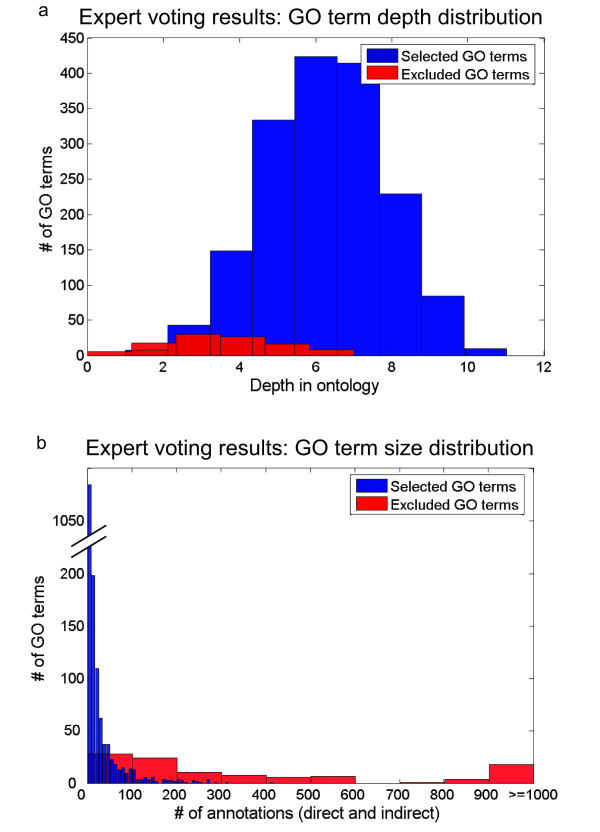
**Depth and size properties of GO terms selected or excluded from the evaluation gold standard based on expert curation**. The functional gold standard based on voting from an expert panel cannot be approximated by either a size or a depth measure of specificity. (**a**) Distribution of GO term depths for expert-selected terms (4–6 votes) and expert-excluded terms (1–3 votes). The selected set of terms cannot be separated from the "too general" excluded terms on the basis of depth. For instance, 53 of the 107 general GO terms appear at depth 4 or lower and 51 of 1692 specific GO terms appear at depth 3 or higher. (**b**) Distribution of GO term sizes (direct and indirect annotations) for the selected and excluded terms based on the expert voting analysis. As with term depth, size cannot effectively distinguish specific terms from those deemed too general by experts. For example, 28 of 107 GO terms deemed too general for inclusion in the standard have fewer than 100 annotations.

Although GO can serve as a good basis for a functional gold standard, effective mapping from organism-specific annotations to a set of positive and negative examples is critical. In particular, we have addressed the problem of varying levels of resolution in the GO hierarchy by selecting the gold standard set of terms through curation by six expert biologists. Through this formal curation process, the experts selected terms that are specific enough to be confirmed or refuted through laboratory experiments while also general enough to reasonably expect high-throughput assays to provide relevant information (see details in Methods and additional file 3: Supplementary discussion). The result of this process is a set of specific functional classes (GO terms) which can be used to generate an accurate set of positively related gene pairs or to directly evaluate or train computational approaches that explicitly associate proteins with particular biological processes. This standard created using expert knowledge is quite different from GO standards commonly used in the literature (Fig. 4). It can serve as a *single*, common standard that addresses the specific concerns of functional genomics.

This curation can also be used to obtain a negative standard which addresses some issues with currently used methods. Specifically, our standard includes a set of negatives more broadly representative than sources such as localization while excluding likely positive examples (a shortcoming of approaches that use random sampling). Further, the standard approximates the correct relative balance of positive and negative sets enabling biologically relevant evaluations (see Methods for details).

#### Evaluating genomic methods and data

In addition to defining a unifying standard, it is critical to use the standard in a manner that accurately reflects the biological reliability of datasets or methods. To expressly address the process-specific variability in accuracy, we developed an evaluation framework that facilitates identification of functional biases in current general evaluations. To accomplish this, we propose that two complementary modes of analysis accompany any evaluation of functional genomic data: (1) a genome-wide evaluation that estimates general reliability but also reports the functional composition of the results and (2) a process-specific evaluation in which the data or method is independently evaluated against a set of expert-selected processes.

##### Genome-wide evaluation

**Figure 5:**
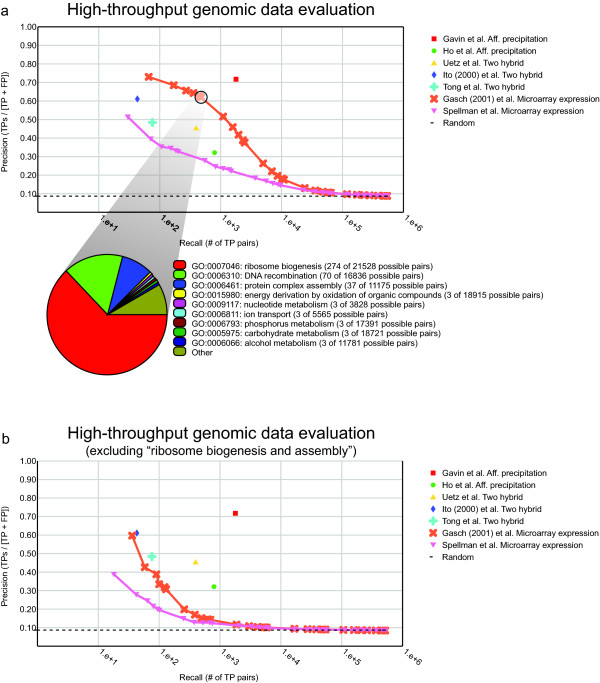
**General (whole-genome) evaluation example**. (**a**) Example of a genome-wide evaluation of several different high-throughput datasets using our framework. These datasets include five protein-protein interaction datasets, including yeast 2-hybrid [16,34,35] and affinity precipitation data [14,36], and two gene expression microarray studies [37,38]. Pearson correlation was used as a similarity metric for the gene expression data. The functional composition of the correctly classified set can be investigated at any point along the precision-recall trade-off, as is illustrated for the Gasch *et al*. co-expression data. This analysis reveals that a large fraction of the true positive predictions (> 60%) made by this dataset are associations of proteins involved in ribosome biogenesis. Of the 500 true positive pairs identified at this threshold, 298 are pairs between proteins involved in ribosome biogenesis, suggesting that the apparent superior reliability may not be general across a wider range of processes. (**b**) The same form of evaluation as in (**a**), but with a single GO term ("ribosome biogenesis and assembly," GO:0042254) excluded from the analysis, a standard option in our evaluation framework. With this process excluded, the evaluation shows that neither of the co-expression datasets is as generally reliable as the physical binding datasets. Additional functional biases can be interrogated through this analysis and corrected if necessary.

To provide a genome-wide analysis that also features information on the constituent biological processes, we have developed a hybrid evaluation framework that combines traditional measures of the precision-recall tradeoff with an analysis of the biological processes accurately represented in the data. In addition to the usual estimation of precision-recall characteristics, we compute the distribution of biological processes represented in the set of correctly classified positives (true positives) at every point along the precision-recall tradeoff curve (Fig. 5). This distribution allows one to identify and measure any biases in the set of positive results toward a specific biological process and interpret evaluation results accordingly. Furthermore, all of this information is summarized and presented in a dynamic and interactive visualization framework that facilitates quick but complete understanding of the underlying biological information.

Figure 5 illustrates an example of a genome-wide evaluation of several high-throughput datasets using our framework. At first glance, a general evaluation indicates that the Gasch *et al*. microarray data is the second most reliable source for functional data (Fig. 5a). However, an analysis of the processes represented in the set of correctly classified pairs reveals that approximately 60% of the correct predictions by the co-expression data are related to the process of ribosome formation (Fig. 5a, bottom chart). This type of analysis is included for any evaluation done with our system and interactive visualization allows for quick and accurate detection of any biases that might be present.

In addition to identifying biases in genome-wide evaluations of datasets or methods, our evaluation framework provides a way to normalize these biases out of the analysis. A user can choose to exclude all positive examples related to one or more biological processes. Figure 5b illustrates an example of this functionality for the evaluation discussed above. Based on the bias we observed, we excluded all proteins involved in ribosome biogenesis and assembly (GO term GO:0042254) and re-evaluated the same set of datasets. While none of the interaction datasets change significantly with this process excluded, both gene expression datasets show substantial decay in their precision-recall characteristics, suggesting they are generally less reliable at predicting functional relationships over a broad range of processes. This result is quite different from what we might have concluded had we not been able to discover and correct this process-specific bias.

##### Process-specific evaluation

Many biological laboratories focus on specific processes or domains of interest, even when using high throughput data/methods. In such situations, a targeted, process-specific evaluation is often more appropriate than a genome-wide evaluation. Our framework facilitates convenient and representative process-specific evaluations by performing independent precision-recall analysis for each process of interest.

**Figure 6:**
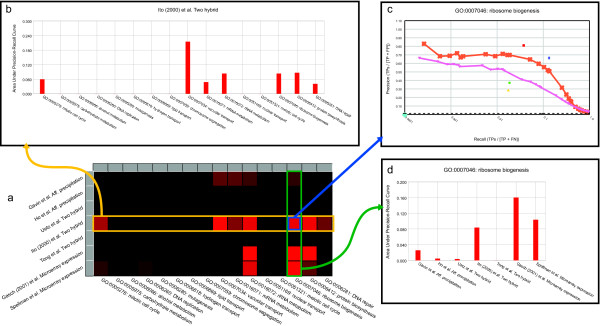
**Process-specific evaluation example**. A detailed understanding of which specific biological signals are present in a particular dataset is important for robust evaluation. Our evaluation framework allows users to query specific processes of interest. (**a**) Example of an evaluation of 7 high-throughput datasets over a set of 16 user-specified processes (GO terms). The precision-recall characteristics of each dataset-process combination were computed independently and the intensity of the corresponding square in the matrix is scaled according to the area under the precision-recall curve (AUPRC). (**b**) Detailed comparison of results for a single dataset, which can be accessed directly from the summary matrix. The AUPRC statistic of a particular dataset (e.g. Ito *et al*. two-hybrid) for each process is plotted to allow for comparison across a single dataset. (**c**) The actual precision-recall curve (from which the AUPRC was computed) is also easily accessible from our evaluation framework. Users can view underlying details of the AUPRC summary statistic which appears in the other three result views. (**d**) The AUPRC results for a single biological process across all datasets can also be obtained from an evaluation result. This allows for direct measure of which datasets are most informative for a process of interest.

For effective presentation of process-specific evaluation results, we have developed an interactive matrix-based view that facilitates comparative evaluation of multiple datasets across several targeted biological processes (Fig. 6). This method allows for easy and dynamic inter-process and inter-dataset comparisons. In addition, precision-recall characteristics for any process are readily accessible, allowing for a more detailed view of the results. Thus, our framework combines general and specific evaluations, enabling accurate interpretation of functional genomics data and computational methods. This community standard can facilitate the comparisons necessary for formulating relevant biological hypotheses and determining the most appropriate dataset or method for directing further experiments.

## Conclusion

We have identified a number of serious issues with current evaluation practices in functional genomics. These problems make it practically impossible to compare computational methods or large-scale datasets and also result in conclusions or methods that generalize poorly in most biological applications. We have developed an expert-curated functional genomics standard and a methodological framework that address the problems we have identified. We hope these can serve as an alternative to current evaluation methods and will facilitate accurate and representative evaluation. Furthermore, we hope our analysis will initiate a broader community discussion about appropriate evaluation techniques and practices.

In recent years, the computational community has played an influential role in the field of genomics by contributing many valuable computational methods that facilitate discovery of biological information from high-throughput data. However, without an accurate understanding of how well the computational methods perform, the role of bioinformatics in directing experimental biology will remain limited. Lack of accurate assessment of the experimental methods themselves hinders both interpretation of the results and further development of genomic techniques. Thus, representative evaluation of computational approaches and high throughput experimental technologies is imperative to our ability as a community to harness the full potential of biological data in the post-genome era.

## Methods

### GO-based functional gold standard

With the Gene Ontology and corresponding annotations in hand, the main issue in generating a standard for evaluation is deciding which terms are specific enough to imply functional associations between gene products. As noted in Results and discussion, the typical approach to this problem has been to select a particular depth in the ontology, below which all co-annotated genes are taken to be positive examples. This has obvious problems in that biological specificity varies dramatically at any given depth in the ontology (see Fig. 3 and Table 1 for details). Another approach reported in the literature is to use term size (i.e. the number of gene product annotations) as a proxy for biological specificity. Using this approach, gene products co-annotated to terms smaller than a certain threshold are considered positive examples. The number of annotation genes, however, is not only a function of how specific a particular term is, but often how well-studied the area is. Thus size is not always an accurate indicator of specificity, and this problem only becomes worse in organisms that are less well-studied.

To address the issue of biological specificity of positive examples, we chose the less automated but more direct and biologically consistent approach of expert curation. For this task, we chose six biological experts with doctorate degrees in yeast genomics. This group contains a cumulative total of more than 40 years of post-doctoral experience working with yeast in a research setting. Instead of using characteristics of the GO term (e.g. depth in the hierarchy, number of annotations) to determine specificity, we instructed our expert panel to formally assess which GO terms are specific enough to imply a meaningful biological relationship between two annotated proteins. More precisely, we instructed the experts to select terms with enough specificity that predictions based on them could be used to formulate detailed biological hypotheses, which could be confirmed or refuted by laboratory experiments. This curation was performed for all GO terms from the biological process branch of the ontology without information of their hierarchical relationships, and each set of resulting responses was corrected for hierarchical inconsistencies. Responses for all experts were then merged by counting the number of votes for each GO term and terms that received more than three votes were selected for the positive evaluation standard. The final counts for all GO terms can be obtained from Biological expert voting results.

Given this set of specific GO terms, we can generate a positive pairwise gold standard by considering all proteins co-annotated to each term as positives. This set of specific functional classes can also be used to directly evaluate or train computational approaches that explicitly associate proteins with particular biological processes as well. For this, we start with the set of specific terms and obtain a non-redundant set by removing any terms whose ancestors are also in the set. This set of terms can be obtained from additional file 2: Non-redundant set of specific GO terms.

We can also use the results of this voting procedure to define a representative set of negative examples. We expect that GO terms receiving 1 or fewer votes are too general to imply meaningful functional relationships between co-annotated proteins. Furthermore, GO terms with a very large number of direct and indirect annotations (i.e. a substantial fraction of the genome) are most certainly too general to imply meaningful functional relationships between co-annotated members. Thus, we obtain a set of gold standard negatives by finding pairs of proteins in which both members have annotations (other than "biological process unknown") but whose most specific co-annotation occurs in terms with more than 1000 total annotations (~25% of the annotated genome) and with one or fewer votes from our panel of six experts. The resulting negative set is more accurate than random pairs of proteins but is still large enough to reflect our understanding of the relative size of functionally related to unrelated pairs in the genome. Furthermore, this set of negative examples is more representative of the presumed distribution of biological negatives than alternate sources of negative evidence such as co-localization. The final gold standard based on this analysis can obtained from : GO-based yeast functional gold standard. This file contains the final pairwise gold standard set of positive and negatives resulting from our expert curation. Yeast protein pairs classified as positives are labeled with a Â“1Â” and pairs classified as negative in the standard are indicated with a -1.

The resulting set of gold standard positive and negative examples is quite different from previously used GO standards based on size or depth as a measure of biological specificity. Figure 4 illustrates this, plotting a histogram of GO term depth and size for both the excluded and included GO term sets based on the biological expert voting procedure described above. Because our gold standard is based on direct re-evaluation of the gene ontology with respect to functional genomics, there are a number of non-specific GO terms excluded based on the voting results that appear relatively deep in the ontology, and conversely, a number of relevant GO terms included that appear near the root (Fig. 4). A similar trend is true of the GO term sizes of the selected and excluded set: many of the GO terms excluded on the basis of expert voting have relatively few annotations. This confirms our earlier observation that neither size nor depth in the ontology serve as good measures of biological specificity. Basing the criteria for generating a GO-based gold standard instead on expert knowledge ensures that the standard is consistent in terms of the biological specificity of the relationships it is capturing and can therefore provide a meaningful basis for evaluation.

Other efforts have previously aimed to derive summary terms from the GO hierarchy, most notably the Saccharomyces Genome Database's (SGD) GO Slim set [19]. This set, however, is not generally appropriate for the purposes of functional evaluation as it was constructed to be a set of "broad biological categories" meant to span the entire range of processes [19]. The functional relationships captured by such broad terms are often too general to provide a meaningful basis for data evaluation. For example, protein biosynthesis (GO:0006412) is one such term included in the GO Slim set, which has approximately 800 annotated genes. A prediction of an uncharacterized protein's involvement in "protein biosynthesis" would not be specific enough to warrant further experimental investigation in most cases. Furthermore, from the perspective of defining an accurate pairwise evaluation standard, clearly not every pair of genes within this set (over 300,000 possible pairwise combinations) has a specific functional relationship.

### Metrics for evaluation: ROC and precision-recall curves

Sensitivity-specificity and precision-recall analysis are two approaches to measuring the predictive accuracy of data from two classes given the class labels (referred to here as positive and negative). Sensitivity and specificity are typically computed over a range of thresholds (for multi-valued data) and plotted with respect to one another. Such an analysis is known as a Receiver Operating Characteristic (ROC) curve and portrays the trade-off between sensitivity and specificity. Each threshold yields one point on the curve by considering protein pairs whose association in the data exceeds the threshold value to be positive predictions and other pairs to be negative. Precision-recall analysis is done in the same way, but with precision (or PPV) replacing specificity. Each of these quantities is calculated as follows:

True positives (TP): protein pairs associated by data and annotated as positives in gold standard

False positives (FP): protein pairs associated by data and annotated as negatives in gold standard

True negatives (TN): protein pairs not associated by data and annotated as negatives in gold standard

False negatives (FN): protein pairs not associated by data and annotated as positives in gold standard

Precision=TPTP+FP,Recall=TPTP+FN
 MathType@MTEF@5@5@+=feaafiart1ev1aaatCvAUfKttLearuWrP9MDH5MBPbIqV92AaeXatLxBI9gBaebbnrfifHhDYfgasaacH8akY=wiFfYdH8Gipec8Eeeu0xXdbba9frFj0=OqFfea0dXdd9vqai=hGuQ8kuc9pgc9s8qqaq=dirpe0xb9q8qiLsFr0=vr0=vr0dc8meaabaqaciaacaGaaeqabaqabeGadaaakeaafaqabeqacaaabaGaeeiuaaLaeeOCaiNaeeyzauMaee4yamMaeeyAaKMaee4CamNaeeyAaKMaee4Ba8MaeeOBa4Maeyypa0ZaaSaaaeaacqqGubavcqqGqbauaeaacqqGubavcqqGqbaucqGHRaWkcqqGgbGrcqqGqbauaaGaeiilaWcabaGaeeOuaiLaeeyzauMaee4yamMaeeyyaeMaeeiBaWMaeeiBaWMaeyypa0ZaaSaaaeaacqqGubavcqqGqbauaeaacqqGubavcqqGqbaucqGHRaWkcqqGgbGrcqqGobGtaaaaaaaa@5338@

Specificity=TNTN+FP,Sensitivity=TPTP+FN
 MathType@MTEF@5@5@+=feaafiart1ev1aaatCvAUfKttLearuWrP9MDH5MBPbIqV92AaeXatLxBI9gBaebbnrfifHhDYfgasaacH8akY=wiFfYdH8Gipec8Eeeu0xXdbba9frFj0=OqFfea0dXdd9vqai=hGuQ8kuc9pgc9s8qqaq=dirpe0xb9q8qiLsFr0=vr0=vr0dc8meaabaqaciaacaGaaeqabaqabeGadaaakeaafaqabeqacaaabaGaee4uamLaeeiCaaNaeeyzauMaee4yamMaeeyAaKMaeeOzayMaeeyAaKMaee4yamMaeeyAaKMaeeiDaqNaeeyEaKNaeyypa0ZaaSaaaeaacqqGubavcqqGobGtaeaacqqGubavcqqGobGtcqGHRaWkcqqGgbGrcqqGqbauaaGaeiilaWcabaGaee4uamLaeeyzauMaeeOBa4Maee4CamNaeeyAaKMaeeiDaqNaeeyAaKMaeeODayNaeeyAaKMaeeiDaqNaeeyEaKNaeyypa0ZaaSaaaeaacqqGubavcqqGqbauaeaacqqGubavcqqGqbaucqGHRaWkcqqGgbGrcqqGobGtaaaaaaaa@5D31@

ROC and precision-recall curves can be summarized with a single statistic: the area under the curve. For ROC curves, we refer to this statistic as the AUC, which is equivalent to the Wilcoxon rank-sum (Mann-Whitney) statistic. Precision-recall characteristics can be summarized with a similar measure which we refer to as the AUPRC. For all plots shown here, we have used AUPRC because precision is more informative than specificity for the typical sizes of positive and negative example sets as discussed in the "Relative size of gold standard positive/negative sets" section of Results and discussion.

### Implementation of web-based evaluation framework

To facilitate community use of the standard, we have implemented our evaluation framework in a public, web-based system available at [13]. All evaluations are based on the standard described in "Defining a new gold standard", which is also available for download at : GO-based yeast functional gold standard and additional file 2: Non-redundant set of specific GO terms. The website allows users to upload genomic datasets for evaluation and includes several widely used high throughput datasets (including those described here) for comparative evaluation. The methods for presenting evaluation results, including all graphs and interactive components, were implemented in SVG (Scalable Vector Graphics), which can be viewed on most browsers with freely available plugins (see Help at [13] for details). The web interface was implemented in PHP, with a back-end MySQL database and C++ evaluation server.

## Authors' contributions

CLM performed the survey of existing evaluation methods, identified the major issues discussed here and devised the evaluation framework to address these problems. DRB and CLM implemented the evaluation website, database and server. MAH and CH provided input on defining a new gold standard for evaluation and key ideas for developing the evaluation framework. OGT proposed the study and supervised the project. All authors read and approved the final manuscript.

## Supplementary Material

Additional File 1 Biological expert voting results. This file contains the results of the voting procedure used to generate a functional gold standard based on the Gene Ontology (described in detail in Methods). Experts selected terms that are specific enough to direct laboratory experiments, but are also general enough to reasonably expect high-throughput assays to provide relevant information. Click here for file

Additional File 2 Non-redundant set of specific GO terms. This file contains a non-redundant set of GO terms receiving more than 3 votes (of 6) from experts. The non-redundant set was obtained by removing any term whose ancestor in the hierarchy is also in the set. Click here for file

Additional File 3 Supplementary discussion. This file contains a more detailed discussion of the relative size of gold standard positive and negative example sets and associated issues. Click here for file

## Figures and Tables

**Table 1:** Example depth five biological process GO terms. GO term depth is a commonly used metric for biological specificity in the Gene Ontology. 5 of the smallest depth 5 GO terms and 4 of the largest depth 5 GO terms are listed above. The processes described range from very specific behaviors (e.g. contractile ring contraction) to less informative groupings (e.g. cellular protein metabolism), suggesting depth is a poor measure of specificity. The size distribution for all depth 5 GO terms is plotted in Fig. 3.

**GO term**	**Term depth**	**Total annotations**
lipoic acid metabolism (GO:0000273)	5	1
cytokinesis, contractile ring contraction (GO:0000916)	5	1
DNA ligation (GO:0006266)	5	1
lysosomal transport (GO:0007041)	5	1
regulation of sister chromatid cohesion (GO:0007063)	5	1
cytoskeleton organization and biogenesis (GO:0007010)	5	285
transcription (GO:0006350)	5	474
protein biosynthesis (GO:0006412)	5	775
cellular protein metabolism (GO:0044267)	5	1381
