# Supplementary material for: Finding function: evaluation methods for functional genomic data
Source: BMC Genomics. 2006 Jul 25;7:187. doi: 10.1186/1471-2164-7-187 (PMC1560386; doi:10.1186/1471-2164-7-187)
Supplement: Additional File 3 — Supplementary discussion. This file contains a more detailed discussion of the relative size of gold standard positive and negative example sets and associated issues. [file 1471-2164-7-187-S3.pdf]

Finding function: evaluation methods for functional genomic data

Chad L. Myers[1,2], Daniel R. Barrett[1,2], Matthew A. Hibbs[1,2], Curtis Huttenhower[1,2], Olga G. Troyanskaya[1,2,†]

[1]Department of Computer Science, Princeton University, Princeton, NJ 08544, USA.

[2]Lewis-Sigler Institute for Integrative Genomics, Princeton University, Princeton NJ, 08544, USA.

[†]To whom the correspondence should be addressed at ogt@cs.princeton.edu

Supplementary Discussion

Relative size of gold standard positive/negative sets calculations

In the manuscript, we quote the sensitivity and specificity statistics for a recently published method, and estimate what these numbers imply for the method's performance on real whole-genome data. We show details of these calculations here.

Estimating the ratio of true positives (TP) to false positive (FP):

If we assume the ratio of interacting protein pairs to non-interacting pairs is approximately 1:20 over the whole-genome, this yields roughly 17.1 million negative protein pairs and 900,000 positives. Thus, a method with a sensitivity of 90% can be expected to detect 810,000 pairs (900,000 * 0.9). The 63% specificity implies a 37% false positive rate (FPR) or ~6.3 million false positive pairs (17.1 million * 0.37). Thus, the ratio of true positive predictions to false positive predictions is ~.13 (810,000 / 6.3 million) or in other words, 1 out of every 9 predictions can be expected to be correct.

Estimating the measured and expected precision:

The method reporting 90% sensitivity and 63% specificity used gold standard negative and positive sets of 2000 and 1500 pairs respectively. 90% sensitivity implies that 1350 true positives were detected by the method (1500 * 0.9). 63% specificity implies a FPR of 37%, suggesting 740 false positives were predicted (2000 * 0.37). Thus, we compute a precision of 65% $\left(\frac{1350}{1350+740}\right)$ on the gold standard.

If we were to apply the same method on a whole-genome scale where the ratio of related protein pairs to unrelated pairs was approximately 1:20, the precision would be much different. Based on our analysis above, which yielded 810,000 true positive pairs and 6.3 million false positive pairs, we estimate a precision of 11% $\left(\frac{810{,}000}{810{,}000+6{,}300{,}000}\right)$. This illustrates that precision critically depends on the relative sizes of the positive and negative standard sets.

A note on estimating precision in a real application setting:

One could imagine using such a correction to extrapolate the expected performance in a biological application given the results of an evaluation against an arbitrarily-sized gold standard. However, we caution against using this approach in general, because these estimates

can be very inaccurate, particularly when the evaluation standard is very small. Such estimates are only accurate when the positive and negative examples are perfectly representative of the actual distribution in the application setting, which is usually not true of specialized standards used in the literature. Thus, the correction described here should only be used as a last resort. Instead, we recommend using a more representative gold standard such as that described in this paper.

Description of biological expert curation process

To select a specific set of GO terms, we asked the help of biological experts. We chose six biological experts, all with doctoral degrees in yeast genomics. These experts have a combined total of more than 40 years of research experience in various facets of yeast biology, including specific topics such as genome stability, enzyme kinetics, metabolism, and nutrient response as well as more general knowledge of functional genomics and bioinformatics. These experts examined 9296 GO terms (all terms under biological process) through a series of forms that allowed them to easily examine the scope of each term and its annotations. They were instructed to consider the question: "if unknown protein P were predicted to be annotated to GO term G, would that be enough to consider experimentally testing this relationship between P and G?". Biologists voted individually (without communication with each other on this process), and voting results were tallied. The experts were not given the hierarchy itself and were asked to assess specificity based on the term's description and annotations. To ensure consistency within each expert's voting results, we forced any children of terms receiving votes for that expert to also receive votes. After forcing consistency, the responses were merged by counting the total number of votes for each term. GO terms receiving 4 or more votes of 6 were selected. The final vote totals for all GO terms can be downloaded additional file 1: Biological expert voting results.
